# Angptl2 is a Marker of Cellular Senescence: The Physiological and Pathophysiological Impact of Angptl2-Related Senescence

**DOI:** 10.3390/ijms222212232

**Published:** 2021-11-12

**Authors:** Nathalie Thorin-Trescases, Pauline Labbé, Pauline Mury, Mélanie Lambert, Eric Thorin

**Affiliations:** 1 Montreal Heart Institute, University of Montreal, Montreal, QC H1T 1C8, Canada; pauline.labbe@umontreal.ca (P.L.); pauline.mury@umontreal.ca (P.M.); melanie.lambert.2@umontreal.ca (M.L.); eric.thorin@umontreal.ca (E.T.); 2 Department of Pharmacology and Physiology, Faculty of Medicine, University of Montreal, Montreal, QC H3T 1J4, Canada; 3 Department of Surgery, Faculty of Medicine, University of Montreal, Montreal, QC H3T 1J4, Canada

**Keywords:** programmed senescence, senescence, angiopoietin-like 2, biomarker, age-related diseases

## Abstract

Cellular senescence is a cell fate primarily induced by DNA damage, characterized by irreversible growth arrest in an attempt to stop the damage. Senescence is a cellular response to a stressor and is observed with aging, but also during wound healing and in embryogenic developmental processes. Senescent cells are metabolically active and secrete a multitude of molecules gathered in the senescence-associated secretory phenotype (SASP). The SASP includes inflammatory cytokines, chemokines, growth factors and metalloproteinases, with autocrine and paracrine activities. Among hundreds of molecules, angiopoietin-like 2 (angptl2) is an interesting, although understudied, SASP member identified in various types of senescent cells. Angptl2 is a circulatory protein, and plasma angptl2 levels increase with age and with various chronic inflammatory diseases such as cancer, atherosclerosis, diabetes, heart failure and a multitude of age-related diseases. In this review, we will examine in which context angptl2 was identified as a SASP factor, describe the experimental evidence showing that angptl2 is a marker of senescence in vitro and in vivo, and discuss the impact of angptl2-related senescence in both physiological and pathological conditions. Future work is needed to demonstrate whether the senescence marker angptl2 is a potential clinical biomarker of age-related diseases.

## 1. Introduction

Cellular senescence is a stress-response mechanism inducing cell cycle arrest; cells cease to divide, but remain metabolically active and undergo distinctive phenotypic changes [1]. There are many causes of cellular senescence, it can occur at any point in life, but only during aging and prematurely with age-related disease will they accumulate. Cells enter senescence due to telomere shortening and activation of the DNA-damage response (DDR) [2,3,4]. Two major pathways trigger and maintain senescence: p53/p21 and p16^INK4a^/pRb pathways [5]. Other mechanisms then reinforce senescence, such as the production of ROS [6] or pro-inflammatory cytokines [7]. Senescent cells are identified using a combination of markers, which include, among others, high levels of p16 and p21, two cyclin-dependent kinase inhibitors (CDKi), detection of lysosomal hydrolase activity at pH 6, also known as senescence-associated β-galactosidase (SA-βGal), identification of senescence-associated heterochromatin foci (SAHF) in the nucleus and loss of nuclear lamina protein lamin B1 (LMNB1) [8,9,10,11].

Senescent cells secrete a plethora of molecules, such as proinflammatory cytokines, chemokines and proteases, gathered within the senescence-associated secretory phenotype (SASP) [12,13]. The composition of the SASP can be influenced by the inducer of senescence, by the cell type that secretes the SASP [14], and as time progresses after senescence induction [15,16]. The main pathways identified as SASP regulators converge to two transcription factors, NF-κB and C/EBPβ, making them the two focal points of SASP regulation [3,16,17]. SASP factors may induce positive (such as tumor suppression, wound healing, regulation of cardiac fibrosis) or negative (such as tumor promotion, degenerative phenotype, atherosclerosis) effects, depending on the context [18]. SASP factors seem to spread from cell to cell, as they act both in an autocrine and paracrine manner [19]. Another important role of the SASP is the recruitment of immune cells to clear senescent cells as well as oncogene-expressing cells [20]. Thus, a transient exposure to SASP factors may be beneficial to allow for remodelling, repairing, attracting macrophages to eliminate cellular and extracellular debris and resolving tissue damage, including clearance of senescent cells to terminate repair [18]. In contrast, chronic exposure to SASP factors is deleterious [21], as the accumulation of SASP factors and senescent cells contributes to chronic inflammation often observed during aging, which might overwhelm the immune system and prevent the clearance of senescent cells [18]. 

Among the numerous SASP factors is angiopoietin-like 2 (angptl2), a protein from the angiopoietin-like family, a family of eight (angptl1–8) members. Angptl2 is mostly known for its pro-inflammatory properties and its contribution in chronic diseases (for review, see [22,23]). It was cloned, expressed and characterised for the first time in 1999 [24]; angptl2 is a glycosylated protein of 493 amino acids (57 kDa) and a secreted circulating protein [24,25,26]. Its structure is similar to that of angiopoietins: it possesses a typical N-terminal helical coiled-coil domain, a short linker peptide and a C-terminal globular fibrinogen-like domain [24]. The former domain could form dimeric or trimeric coiled-coils and enhances the survival of hematopoietic stem cells [27], whereas the fibrinogen-like domain is thought to be a putative receptor binding site [28]. Depending on the cell type, angptl2 binds to different receptors: in adipocytes and endothelial cells, angptl2 binds to integrins α5β1 [26,29]. In hematopoietic cells [30], platelets [31] and in some pancreatic cancer cells [32] the immune-inhibitory receptor human leucocyte immunoglobulin-like receptor B2 (LILRB2) and its mouse orthologue paired immunoglobulin-like receptor (PIRB), have been reported to bind angptl2. Recently, in adipocytes, CD146 was reported to be a new receptor for angptl2 [33]. Finally, in kidney cells, intracellular angptl2 binds to the intracellular C-terminal domain of AT1A receptors [34,35]. No specific antagonist or inhibitor of angptl2 receptor has been designed. To inactivate angptl2, only antisense, small or micro interference RNA or genetically modified angptl2 knockout/knockdown mice approaches are used in preclinical studies (for review, see [22,23,36]). In humans, anti-diabetic drugs [26] and statins [37], in addition to lifestyle changes such as physical training [38,39] and/or diet [40] have been shown to reduce circulating angptl2 in patients with age-related diseases. 

Angptl2 is a multifaceted protein with both physiological and pathological functions (for review, see [22,23]), similarly to other SASP factors. Angptl2 was originally acknowledged for its proangiogenic [24,41,42] and antiapoptotic capacities [28]. Beneficial angiogenic properties of angptl2 were reported in the context of stroke [43], and one study demonstrated that angptl2 was antithrombotic [31]. Angptl2 may also contribute to vasculogenesis [44]. Importantly, angptl2 increases the survival and expansion of hematopoietic stem and progenitor cells [45,46,47] (for review, see [48]). Angptl2 may also be a key player in intestinal stem cells by regulating intestinal epithelial regeneration [49]. Angptl2 protects against lung fibrosis [50], promotes beneficial innate immune response [51] and also contributes to cell differentiation in osteoblasts [52]. Globally, by promoting adaptive inflammation and tissue reconstruction, angptl2 may maintain tissue homeostasis (for review, see [22]). Nonetheless, circulating angptl2 levels, very low in healthy active young individuals [36,53], rise gradually with age in the general population [29]. Angptl2 is thus better acknowledged for its association with multiple chronic diseases, in particular in various types of cancers (for review, see [36]). Furthermore, increased levels of angptl2 were reported in diabetes, chronic kidney disease, cardiovascular diseases, metabolic disorders including obesity, and other diseases (for review, see [22,23,36]). Angptl2 may contribute to the pathogenesis of these chronic inflammatory and age-related diseases via its inflammatory [26], pro-fibrotic [54], pro-oxidant properties [55,56] and activation of metalloproteinases [57] (for review, see [36]). 

Another property of angptl2, rarely mentioned in the literature, is its contribution to cellular senescence. Despite the fact that angptl2 has been identified in a multitude of SASP signatures and in various cell types exposed to different stressor stimuli, its exact role in the context of senescence is usually not discussed. However, angptl2 shares striking functional similarities with typical SASP proteins. Indeed, the physiological and pathological functions of angptl2 share multiple connections with various effects of cellular senescence. In addition, circulating levels of angptl2 are used for the prediction and prognosis of multiple age-related diseases (for review, see [36]), in which cellular senescence and SASP are key players. To the best of our knowledge, there is no literature clearly establishing that angptl2 is a marker of senescence and there is no review gathering the data concerning the potential role of angptl2-related senescence in physiological or pathophysiological conditions. In this review, we will therefore examine different studies in which angptl2 was identified as a SASP factor, discuss the experimental evidence showing that angptl2 is a marker of senescence in vitro and in vivo, and debate the impact of angptl2-related senescence in both physiological and pathological conditions. We propose that angptl2 is an understudied senescence marker with a potential—but still speculative—clinical utility as a biomarker of age-related diseases. Whether angptl2 could be a therapeutic target is beyond the scope of this review.

## 2. Angptl2 Is a Marker of Cellular Senescence

The first report listing angptl2 in the SASP of senescent fibroblasts was published in 2003 [58]. At that time, the function of angptl2 was mostly unknown, and its role as a senescent marker was not discussed. In fact, until 2008, angptl2 was only known as a pro-angiogenic, an anti-apoptotic and a growth factor, as mentioned above. To the best of our knowledge, our laboratory was the first to point out a specific association of angptl2 with senescence [59] in cultured endothelial cells isolated from atherosclerotic patients. 

### 2.1. Identification of Angptl2 in Senescent Cells

**Figure 1:**
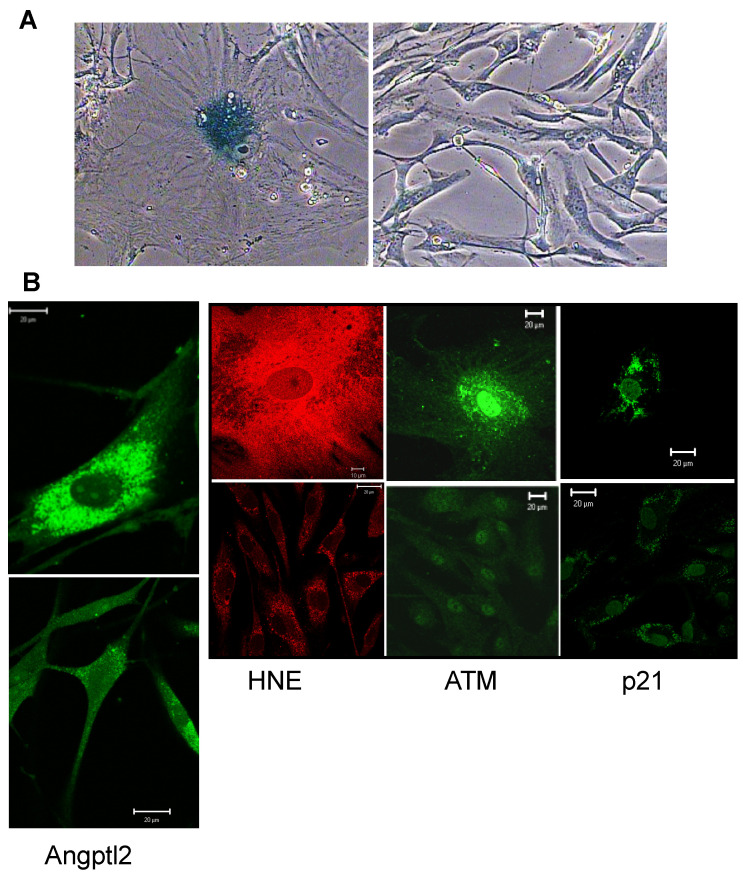
Angptl2 expression in human senescent endothelial cells. (**A**) Senescence-associated β-galactosidase staining of senescent (left panel) compared to non-senescent (right panel) cultured hIMAEC; same magnification ×400 (adapted from [61]). (**B**) Immunofluorescence images of senescent (top panel) compared to non-senescent (bottom panel) cultured hIMAEC for angptl2 (adapted from [53]), the oxidative stress marker HNE (adapted from [61]), the DNA damage marker ATM (adapted from [62]) and the senescent marker p21 (NTT, unpublished data). ATM: ataxia-telangiectasia mutated; hIMAEC: human internal mammary artery endothelial cells; HNE: hydroxy-nonenal.

We previously reported that human endothelial cells cultured from discarded segments of the internal mammary artery (hIMAEC) during coronary artery bypass graft (CABG) surgeries massively express and secrete angptl2 [53], senesce rapidly due to a combination of telomere shortening and oxidative stress [60,61], and exhibit a high level of oxidative stress [61] and DNA damage [62] (Figure 1). These cells express typical cell damage markers observed during senescence (for review, see [15]): indeed, in senescent hIMAEC, oxidative damage triggers characteristic DNA double-strand breaks, which promote DDR with the activation of ataxia-telangiectasia mutated (ATM), followed by histone H2AX phosphorylation and p53-dependent promyelocytic leukemia (PML) body accumulation [60]. Although it is unknown whether angptl2 directly induces DNA damage, in the context of cancer, angptl2-associated inflammation creates a microenvironment that causes DNA damage and genomic instability [55]. Angptl2 also lowers the expression of DNA repair enzyme Msh2 by epigenetic methylation [56]. In addition, angptl2 stimulates oxidative stress [53,56]. Angptl2 expression is clearly abnormally high in these senescent hIMAECs characterized by both DNA damage and oxidative stress (Figure 1). Accordingly, we showed that oxidative stress-induced premature senescence observed in hIMAEC from active smokers was associated with higher (4-fold) levels of angptl2 mRNA [59]. After chronic treatment of these cells with the antioxidant N-acetyl cysteine, the expression of angptl2 mRNA was reduced [53] and senescence was delayed [60,61]. 

Angptl2 has been listed as a member of the SASP in multiple other senescent cell types, such as fibroblasts, vascular smooth muscle cells, hepatocytes, astrocytes, platelets, and some cancers cells (Table 1). The molecular mechanisms underlying SASP-derived angptl2 secretion are mostly unknown. 

### 2.2. Transcriptomic and Proteomic Analyses That Identified Angptl2 as a SASP Factor

Before the notion of SASP emerged, a number of genes were shown to be differentially expressed during replicative senescence in human diploid fibroblasts in culture, and an initial catalogue of 28 differentially expressed genes was built [81]. A few years later, the expression of genes coding for matrix-degrading proteases, inflammatory chemokines and cytokines was confirmed in the culture media of senescent cells [82]. The concept of a senescent secretory phenotype was then proposed by the Campisi group [83], known as the SASP [12,13]. Using transcriptomic or proteomic approaches, successive studies of the secretome of senescent cells of different cell types and with various senescence inducers added progressively new members to the SASP [12,76,84,85]. These studies revealed the complexity and the heterogeneity of the SASP, and functional enrichment analyses demonstrated that clusters members of the SASP are involved in diverse biological processes (for example, [86,87]). More recently, single-cell RNA sequencing permitted the analyses of SASP factors on a large scale from cultured senescent cells, and from different tissues isolated from animals at different ages (for review, [88]). It is important to note that there is no exhaustive list of the members of the SASP, but different “SASP atlases” have been recently proposed [78,86], and these atlases are regularly enriched with new SASP factors. 

The angptl2 gene ID is 23452 (gene database) and its UniProt ID is Q9UKU9. The first study that identified angptl2 in the culture medium of senescent cells was that of Zhang et al. [58]: in serially passaged human primary senescent fibroblasts, angptl2 appears in the list of “proliferative-arrest associated genes” (Table 1). The function of angptl2 was then mostly unknown, but functional enrichment analysis associated angptl2 to developmental processes [58]. The numerous studies in which angptl2 was identified in senescent cells as a SASP factor are summarized in Table 1. Regardless of the cell type (fibroblasts, vascular smooth muscle cells, endothelial cells, cancer cells, astrocytes, platelets, mesenchymal stem cells) and the senescence inducer (serial passages, oxidative stress, DNA-damaging drugs, X-ray irradiation), angptl2 expression either increased or decreased in senescent cells (Table 1). Data from Table 1 do not permit an estimation of the homeostatic range of limits of angptl2 since results from *omics* analysis only report fold changes of expression against a well-chosen reference (i.e., housekeeping gene in qPCR experiments, control condition in microarray and RNA sequencing experiments or expression in cells from all other cell types in single-cell RNA sequencing experiments). Nevertheless, Table 1 is informative about the currently available data on angptl2 expression and could help for future analysis focused on angptl2’s role in senescence-associated conditions.

Angptl2 is not generally considered as a major SASP gene or SASP protein but is rather hidden among hundreds of other molecules listed in the supplemental tables enumerating the numerous molecules differentially expressed in the culture medium of senescent cells versus non-senescent cells (Table 1). In some studies, however, angptl2 is clearly identified as a senescence marker: microarray analyses identified up-regulation [72] or down-regulation [71] of angptl2 mRNA expression in senescent hepato-carcinoma cells. In the study of Nagano et al., novel genes, including angptl2, that are specifically upregulated in senescent cells were identified; nevertheless, angptl2 was only mentioned to be a secreted polypeptide, and its function was not discussed [72]. The premature up-regulation of senescence-associated genes, including angptl2, was also reported in fibroblasts from Werner’s syndrome patients—a progeria syndrome—compared with normal healthy fibroblasts [67,89]. The function of angptl2 in this particular pathological context was, once again, not discussed [67]. In contrast, microarray and deep sequencing of human senescent platelets revealed that angptl2 was upregulated and part of a cluster of genes relevant for vascular remodelling and inflammation [80]. Pro-inflammatory genes, including angptl2, were also upregulated in human senescent astrocytes [74,75]. Finally, more recently single-cell RNA sequencing of aged mouse brains listed angptl2 as one of several senescence effector genes in brain microvascular endothelial cells [77]. This list of senescence effector genes, which includes angptl2, was compiled on the basis of a meta-analysis of transcriptomic profiles [90] combined with publicly available whole transcriptome data sets [68,69,90,91]. Angptl2 was also identified by single-cell RNA sequencing in cultured human senescent fibroblasts [66,70] and in human cultured endothelial cells following targeted inactivation of FOXO3A that recapitulates the major phenotypic defects observed in the arteries of aged monkeys [78] (Table 1). A recent informative review of the aging/senescence-related single-cell dataset analyses is available [88].

The SASP proteins were characterized in 2008 using an antibody array [12] targeting 120 particular proteins; angptl2 was not among them, but at that time, angptl2 was mostly unknown. Subsequently, unbiased proteomic studies identified the secretory profile of senescent fibroblasts, according to the inducer, in human cultured cells [63,92] and tissues [79,93]. The first identification of angptl2 within thousands of other SASP proteins was performed in human senescent fibroblasts in culture [63]. Angptl2 was also listed in senescent bone marrow and adipose mesenchymal stromal cells [87]. Recently, new unbiased mass spectrometry analyses have identified new or understudied SASP factors (defined as proteins secreted at significantly different levels by senescent cells), and a SASP atlas was built [86]; angptl2, however, was not listed in this SASP atlas. Based on the literature, another cellular senescence database was compiled [94]; although angptl2 was not listed among the 279 selected human genes driving cellular senescence, this database quoted a meta-analysis [79] in which angptl2 was mentioned in the cellular senescence signature. This latter study compared genes differentially expressed with age and genes differentially expressed in cancer among nine human tissues: the angptl2 gene was expressed in aged prostate and uterus, and it was also expressed in tissues from breast, uterus and brain cancers [79] (Table 1). 

In summary, numerous microarray analyses identified angptl2, among other mRNAs/proteins, as being differentially expressed in senescent cells. To date, however, the impact of senescent cells on angptl2 expression is not clear: in a same-cell type or tissue, an increase or a decrease in angptl2 gene expression has been reported (Table 1). Future single-cell RNA seq experiments focused on age-related pathologies should be of great interest to elucidate the main function of SASP factors (including angptl2) by identifying upstream and downstream regulators and effectors in specific cell types. These high-throughput sequencing technologies will open new avenues in our comprehension of the role of cell senescence effectors, including angptl2, which remains an understudied SASP factor.

### 2.3. In Vivo Experimental Evidence That Angptl2 Is a Senescence Marker

In addition to the numerous evidence given by transcriptomic and proteomic signatures of cultured senescent cells, three recent mouse models demonstrate the contribution of angptl2 to cellular senescence in vivo. First, in skeletal myocyte-specific angptl2 knockout mice (*Angptl2*^Flox/Flox^; *MCK-Cre*), angptl2 suppression improved cellular senescence phenotypes and decreased inflammation and oxidative stress [95]. The expression of classical senescent markers, such as p21, the cytokine IL-6, and SA-βGal, was significantly reduced in the skeletal muscle of *Angptl2*^Flox/Flox^, *MCK-Cre* mice, but also in C2C12 myoblasts transfected with angptl2 siRNA when compared to control C2C12-siRNA cells. The authors proposed that angptl2 might accelerate muscle cell senescence by promoting both inflammation and oxidative stress [95]. Second, in order to study the contribution of telomere length to cardiac valve disease, a mouse Notch1^+/−^ combined with the lack of the telomerase RNA component Terc (*Notch1*^+/−^; m*Terc*^−/−^) was developed [96]. In this mouse model of premature human aging and valve disease, high angptl2 gene expression was reported [96], reinforcing the role of angptl2 in senescence. Third, we showed that in LDLr^−/−^, hApoB100^+/+^ atherosclerotic mice, with vascular delivery of a small hairpin (sh)-angptl2 using an adeno-associated virus serotype 1, an ~80% reduction in *angptl2* mRNA aortic endothelial expression was associated with a concomitant decreased expression of the senescent marker *p21* and other SASP factors (*Pai-1*, *Icam1*, *Mcp1*) [97]. In this mouse model of atherosclerosis, angptl2 down-regulation not only decreased markers of senescence and lowered monocyte recruitment and inflammation in the endothelium, but it also stimulated endothelial repair and slowed atherogenesis [97], demonstrating that angptl2^+^/p21^+^ vascular senescent cells contribute to the atherogenic process. 

We recently confirmed the fact that angptl2 is also a clinical marker of senescence: in human IMA segments from atherosclerotic patients undergoing CABG surgery, *ANGPTL2* mRNA expression strongly and positively correlates with that of *p21* mRNA [97], suggesting that angptl2 is a marker of aging and arterial senescence load. Accordingly, we also reported that circulating angptl2 levels reflect arterial inflammation and senescence, since angptl2 plasma levels decreased after cardiac surgery only in younger patients with lower levels of tissue inflammation and arterial senescence load [98]. 

In summary, angptl2 is secreted by senescent cells, in cultured cells exposed to various stressors, and in vivo in the context of increased demand of repair of cellular damages associated with aging and age-related diseases, both in animal models and in humans; this qualifies this multifaceted protein as a senescent marker. Remarkably, as discussed in the next section, the numerous functions of angptl2 are strikingly similar to those of classical SASP factors, beyond inflammation and irreversible growth arrest.

### 2.4. Similar Functions of the SASP and of Angptl2

Once the persistent DDR cascade signalling is activated, the expression of SASP factors is under the control of transcription factors such as NFκB [99,100] and C/EBP [85] (for review, see [2]). Key SASP components, such as the cytokines IL-6 and IL-8, have been described to be NFκB-dependent pro-inflammatory proteins [21]. Similarly, NFκB controls the transcriptional regulation of angptl2, at least in endothelial cells [29] and in cancer cells [101]. In addition, angptl2 activates NFκB translocation to the nucleus by increasing the degradation of inhibitor κB (IκB) [22,26], resulting in NFκB-dependent inflammatory gene expression, including angptl2 itself in a vicious circle, a phenomenon observed in different cell types (for review, see [23,102]). TGFβ is also a known promoter of senescence via multiple signalling pathways (for review, see [103]); angptl2 activates the TGFβ-Smad cascade in the context of cancer [55] and of mechanical stress [104]. Angptl2 is also a target gene of the TGFβ-Smad transcriptional machinery [105]. Thus, as observed for the canonical SASP cytokines IL-6 and IL-8, angptl2 not only contributes to the SASP, but may also help in maintaining senescence by an autocrine positive feedback loop. 

**Figure 2:**
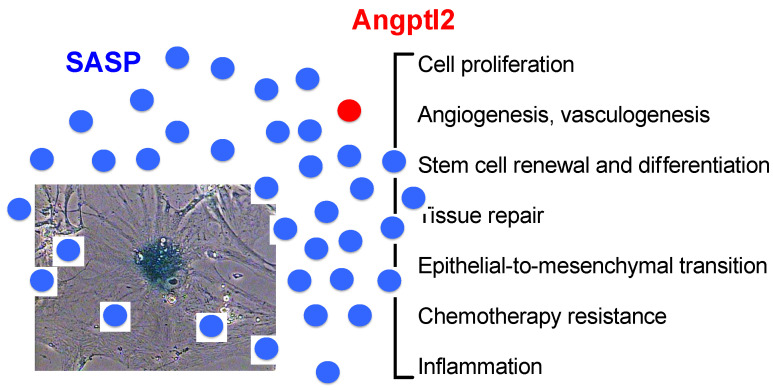
Similar functional characteristics of the SASP and of angptl2. Adapted from [2].

With their constitutive expression becoming permanent with the accumulation of senescent cells with age, many SASP factors, including angptl2, directly or indirectly promote an inflammation that becomes chronic [7]; this is a key contributor to numerous age-related diseases including cardiovascular diseases (for review, see [106]). Similarly, angptl2 is mostly known for its pro-inflammatory properties, first described in the context of obesity and insulin resistance in humans and mice [26], and is implicated in multiple age-related chronic inflammatory diseases [23,36,102] (see below). Beyond inflammation, however, the myriad of factors that compose the SASP displays numerous biological activities (for review, see [2,18]) (Figure 2). Interestingly, a single SASP factor may display both physiological and damaging properties, and this is also the case for angptl2 [22,23].

One beneficial property of the SASP, illustrated during wound healing, is to stimulate cell proliferation, angiogenesis and the formation of new blood vessels through the secretion of growth factors such as VEGF [107]. Angptl2 belongs to the angiopoietin family and was first described as a pro-angiogenic factor [24,41,42]. Angptl2 also positively regulates vasculogenesis by promoting cell migration in in vitro models [44], and controls intestinal epithelial regeneration in mice [49]. Among other genes, angptl2 was also found to be upregulated in the angiogenic repair mechanism occurring after ischemic stroke in rats [43], a disease for which cellular senescence—in particular short telomeres—is suspected to play a role in its aetiology [108,109,110]. Accordingly, a recent clinical study (Senescence-Associated Systems diagnostics Kit for cancer and stroke, SASkit) was proposed to define senescence-associated markers of ischemic stroke [111]. 

Another beneficial property of the SASP is stem cell renewal and differentiation (for review, see [2]), a property also shared with angptl2, which is required for the survival and the expansion of bone marrow-derived hematopoietic stem cells [30,45,46,47,48,112,113] and human cord blood hematopoietic progenitors [27]. Thus, although it is a member of the SASP, angptl2 is also a growth and a survival factor.

SASP factors are beneficially involved in tissue repair, wound healing or tissue regeneration (discussed in the next section); likewise, angptl2 contributes to tissue repair by inducing tissue remodelling in coordination with MMPs activity (for review, see [22]), the latter being typical members of the SASP. Angptl2 also contributes to skin regeneration in fish [114], to fin regeneration in zebrafish [28], and to appendage regeneration in *Xenopus laevis* [115]. Finally, angptl2 is expressed in chondrocytes during bone repair after a fracture [116], contributing to wound healing. 

Although cellular senescence and growth arrest are an anti-tumor mechanism, SASP factors paradoxically also promote tumor progression and cancer [2,117,118,119,120]. Angptl2 is also a biomarker for the diagnosis and prognosis of various types of cancer (for review, see [23,36]). Angptl2 contributes to the inflammatory environment that facilitates carcinogenesis and metastasis by promoting epithelial-to-mesenchymal transition, tumor migration, tumor angiogenesis, and lymph-angiogenesis [32,55,101,121,122,123,124,125]. In contrast, angptl2 has also been proposed to be a tumor suppressor in primary ovarian cancer tissues [126], although recent studies demonstrated that, depending on what cell type angptl2 was expressed in, it could either promote or slow tumor progression [127,128]. 

Some SASP factors secreted by senescent cells can either induce chemotherapy resistance or chemotherapy sensitization to the neighbouring tumor cells (for review, see [2]). It has been shown that angptl2 renders colorectal cancer cells resistant to chemotherapy by activating anti-apoptotic signalling, increasing cancer cell viability and antineoplastic drug resistance [129]. The latter study demonstrated that angptl2 induced chemotherapy resistance in cancer cell lines, but the authors also observed a correlation between higher angptl2 expression in tumors from colorectal cancer patients who underwent chemotherapy, with a lower objective response rate [129]. 

Altogether, these data suggest that SASP factors, including angptl2, can both promote or suppress tumor progression depending on complex cellular and molecular interactions, environment, and still unknown factors. Remarkably, angptl2 alone recapitulates many of the beneficial and deleterious functions of different SASP factors, from tissue repair, stem cell renewal, and differentiation, to cancer progression (Figure 2). Of note, although angptl2 is an identified member of the SASP, its multiple functions have not been well characterized in senescent cells. However, in the context of tissue repair or cancer, the presence of senescent cells and thus of senescent cell-derived angptl2, is very likely. Senescence, like cellular replication or apoptosis, can occur at any point in life, in various physiological and pathological contexts [130].

## 3. Physiological Roles of Angptl2-Related Senescence

### 3.1. Potential Involvement of Angptl2-Related Senescence in Embryogenesis and Development

Although senescence has been mostly and extensively investigated as a response to a stressor in the context of aging, few studies have introduced the unexpected concept of programmed cellular senescence as a crucial mechanism to pattern specific regions and balance cell populations in many tissues of the developing embryo (for review, see [131]). Indeed, the involvement of p21-induced programmed senescence, without DNA damage and in a p53-independent manner, has been demonstrated during normal mammalian development [132,133,134]. Even if the mechanisms surrounding developmental cell senescence are not clearly understood yet, it has been shown that these senescent cells could ultimately either be removed, or loose key senescent marker expression and re-enter the cell cycle, to escape from cell death and clearance, and contribute to organ maturation after birth [135]. 

Previous studies underlined the potential role of angptl2 in embryogenesis. The homologous gene to mammalian *angptl2* is expressed in zebrafish during embryonic development [136]. Functional enrichment analysis using Metascape (http://metascape.org, accessed on 24 August 2021; [137]) showed that heart development was among the top 20 functions enriched with *angptl2* co-expressed genes [138], and GWAS recently identified *angptl2* as a gene related to growth and development in bovines [139]. Although it is still unknown if angptl2 directly participates in embryonic senescence, some evidence supports its potential involvement, particularly in the heart: intense areas of senescence have been identified in developing chicken hearts from E5 to E8 and in mouse embryos at E13.5 (particularly in the outflow tract, a transient structure whose remodelling gives rise to the future aortic and pulmonary valves) [140]. Interestingly, angptl2 is strongly expressed at E4 in the outflow tract of the chicken embryo [141]. In mouse embryos, the public database Eurexpress (www.eurexpress.org, accessed on 24 August 2021; euxassay_007716) also showed the expression of *angptl2* mRNA in aortic and pulmonary valves at E14.5. The concomitant occurrence of strong senescence and increased angptl2 expression in the same areas reinforces the hypothesis that angptl2 could be involved in embryonic organogenesis through the regulation of a programmed senescence pathway. Recently and in accordance with these previous studies, we observed that the aortic valve leaflets of *angptl2*-knockdown mice are prone to dysregulated programmed cell senescence, with a decrease in p21 expression during valve remodelling at embryonic stage E14.5, leading to a premature thickening of the aortic valve at E18, and the development of a spontaneous aortic valve stenosis in adult mice [142]. In addition to contributing to heart development, programmed cell senescence has also been shown to contribute to normal osteogenesis [143]; interestingly, *angptl2*-knockout mice show impaired differentiation of immature chondrocytes during bone formation [116]. Altogether, these results strongly suggest a role of angptl2 in embryonic development by triggering and/or contributing to a programmed senescence response pathway; further studies, however, are required to confirm this hypothesis. 

### 3.2. Physiological Impact of Angptl2-Induced Senescence in Tissue Regeneration and Repair

Programmed cell senescence also occurs through our lifespan after embryonic development. Indeed, while excessive senescence in mature tissues and organs can be harmful, transient localized senescence has been demonstrated to be essential for wound healing and tissue regeneration (for reviews, see [18,144,145]). Some studies pointed out the role of angptl2 in wound healing and tissue regeneration (for review, see [22,23]), without clearly uncovering the link between angptl2 and senescence in this context. Nonetheless, some evidence indicates that angptl2 could contribute to tissue regeneration and repair using senescence as an intermediate step. Angptl2 is involved in the tail regeneration of amphibians [115,146], skin regeneration in sea bream [114], and fin regeneration in *zebrafish* [136], processes for which cell senescence has been clearly shown to contribute [147,148]. In mammals, angptl2 is involved in epithelial wound healing in the intestine after injury [49]. Epithelial regeneration requires mechanisms such as epithelial-to-mesenchymal transition, cell migration, formation of new vasculature, and extracellular matrix remodelling, in which senescence is or could be involved [145,149,150], and in which angptl2 has already been demonstrated to be essential [22,23]. In accordance with this hypothesis, the SASP—which includes angptl2—participates in wound repair: it accelerates cutaneous wound healing through the secretion of growth factors [151] and promotes cell plasticity and stem cell marker expression in keratinocytes and in the liver [152]. While SASP markers increase in the fracture callus and may contribute to improving fracture healing in mice [153], angptl2 expression has also been found to be increased during bone repair after fracture [116]. 

Altogether, this evidence strongly suggests that angptl2 could promote tissue repair via its SASP properties, but further studies are needed to validate this hypothesis. 

## 4. Pathological Impact of Angptl2-Related Senescence

The first direct evidence that cellular senescence causes age-related diseases was provided by an elegant study using the transgenic mouse model INK-ATTAC, in which senescent cells positive for p16^INK4a^ can be eliminated by activation of apoptosis in response to administration of AP20187 [154]. The removal of senescent cells in these INK-ATTAC mice delayed the progression of age-related phenotypes and increased their lifespan and health span [154,155,156]. As highlighted before, chronic low systemic inflammation is typical for aging and is magnified in age-related diseases; SASP factors secreted by senescent cells are, at least partly, responsible for this “inflamm-aging” (for review, see [157]). Indeed, the implantation of senescent fibroblasts in young mice activates an immune response for the targeting and elimination of the senescent cells, a response that is incomplete in old mice where impaired immunity results in inefficiency to destroy the senescent cells, and thus contributes to chronic inflammation [158]. Similarly, transplanted senescent fibroblasts into the knee of mice induce an osteoarthritis-like phenotype, an age-related chronic inflammatory disease [159]; likewise, injection of senescent pre-adipocytes in mice number-dependently induces age-related diseases [160]. Thus, through inflammation and SASP, senescent cells actively contribute to age-related conditions; in addition, senescence begets senescence by a bystander effect [161].

**Figure 3:**
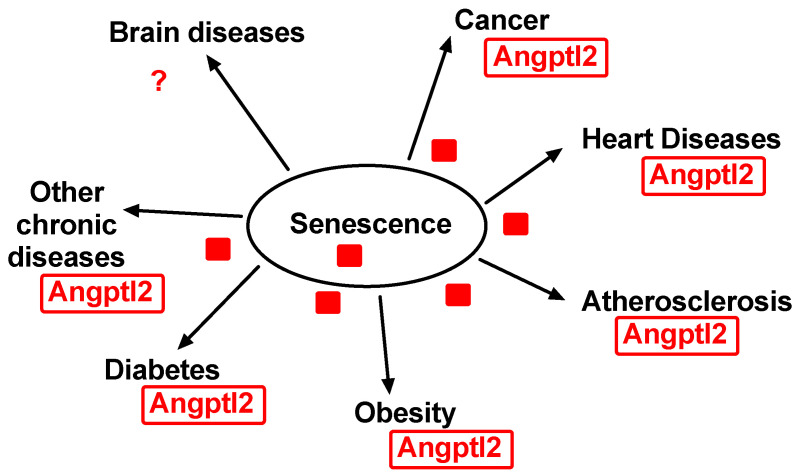
Senescence-related diseases. Angptl2 contributes to or is associated with all chronic diseases related with senescence, such as cancer, cardiovascular diseases, obesity and diabetes, and many other chronic diseases. The role of angptl2 in brain diseases and neurodegenerative diseases is less clear.

Mammals are evolutionarily equipped to deal with acute inflammation synonymous with repair, not with chronic inflammation synonymous with damage [162]; conversely, the elimination of senescent cells protects from age-related diseases (for review, see [10,163]). Multiple excellent reviews already discussed the deleterious contribution of senescence in chronic diseases, such as cancer [119,120,164], heart diseases [165,166], atherosclerosis [167,168,169], obesity [170], type 2 diabetes [171], brain aging and neurodegenerative diseases [172], and other chronic diseases such as congestive obstructive pulmonary diseases (COPD) [173], for example (Figure 3). 

Although angptl2 directly or indirectly contributes to all these chronic diseases (for review, see [23,36,102] (Figure 3)), the exact role of angptl2-related senescence in these diseases has not been clearly defined. As mentioned above, angptl2 has rarely been studied in the context of cellular senescence, but there are a few exceptions. 

First, because circulating levels of angptl2 increase with age [29] and predict cardiovascular diseases [174], diabetes [175], and kidney diseases [176] in the general population, angptl2 was a candidate biomarker in the oldest individuals (from 85 to over 110 years old) [177]. Despite its predictability for these cardiometabolic diseases among the general population, angptl2 was not associated with increased mortality in centenarians and super centenarians [177]. However, because this study included subjects with an exceptional lifespan and health span, the authors suggested that centenarians could exhibit “resistance” [177] or better defence against senescence in general, and angptl2-mediated inflammation in particular. In contrast, circulating angptl2 levels predicted major adverse cardiovascular events and all-cause death in patients with type-2 diabetes and renal dysfunction [178]; in the latter study, senescence was not evaluated.

Secondly, we reported that targeted in vivo elimination of senescent vascular angptl2^+^/p21^+^ cells with an AAV1-shRNA-anti-angptl2 in young pre-atherosclerotic mice significantly reduced the development of the atherosclerotic plaque [97]. Senescent angptl2^+^ cells were eliminated by apoptosis; this favoured the recruitment of endothelial progenitor cells, limiting vascular inflammation and macrophage infiltration and delayed atherogenesis [97]. In a previous study, we conversely reported that angptl2 (not angptl2-induced senescence, which was not evaluated at the time) promoted atherosclerosis, activated leukocyte adhesion to the native endothelium, and induced vascular inflammation in mice [53]. Thus, these data strongly suggest that angptl2-related senescence contributes to atherosclerosis, at least in mice. In humans with risk factors for cardiovascular disease, we reported that circulating levels of angptl2 were abnormally high and correlated with the senescent and inflammatory status of the patients [53,98]. Although indirect, this suggests that angptl2-related senescence also contributes to atherosclerosis in humans.

Third, we have indirect evidence suggesting an association of angptl2-related senescence with severe obesity: in patients undergoing bariatric surgery, the relatively low reduction in angptl2 circulating levels after the surgery (0% at 6 months, 18% at 1 year) did not reflect the drastic weight reduction (27% at 6 months, 37% at 1 year) [179]. This could suggest that damaged/senescent adipocytes, a major source of angptl2 [26,36], are still present months after the surgery—which, per se, is not meant to be senolytic but to limit caloric intake—and may continue to produce angptl2; a longitudinal biopsy-based histological confirmation is necessary to validate this hypothesis. Conversely, another study reported that in overweight and obese subjects without cardiovascular diseases, and thus those less likely to involve senescent adipocytes, a 12-week diet reduced both body weight (−7%) and circulating levels of angptl2 (−15%) [40].

Fourth, we observed that angptl2 gene expression was significantly higher in premature senescent hIMAEC isolated from active smokers, and even higher in senescent cells from patients with COPD when compared to non-smokers [59]. Interestingly, premature stress-induced senescence and inflammatory SASP factors have been proposed to contribute to COPD; indeed, patients with COPD display a “COPD-associated secretory phenotype” reminiscent of the SASP [173].

Finally, angptl2 is associated with many age-related pathologies to which cellular senescence has been shown to contribute, such as rheumatoid arthritis [180], osteoarthritis [181,182,183], gout [184], sarcopenia [95], macular dysfunction and visual impairment [185,186], atherosclerosis [29,53,187], heart diseases [102,188,189], kidney diseases [54,176,190,191], and cancer [192,193]. In brain diseases, the role of angptl2 is less studied [43,194], although angptl2 binds to human leukocyte immunoglobulin-like receptors LILRB2 [30], which are also the receptors for β-amyloid, the hallmark of Alzheimer’s disease [195]. Nevertheless, direct evidence of the causal contribution of senescence-derived angptl2 in age-related diseases, besides atherosclerosis [97], are ill defined.

## 5. Clinical Utility of Angptl2, a Potential Senescent Biomarker of Age-Related Diseases 

A recent study created a proteomic signature of plasma aging markers in a cohort of relatively healthy subjects and found an enrichment of SASP proteins based on reports from the literature [196]. From the 217 age-associated proteins identified in this study, 72 proteins were previously reported as SASP proteins [196]. Conversely, another study showed that the SASP contains age- and disease-related biomarkers: the authors built a useful SASP atlas, identifying several protein markers of senescence that overlap with human plasma markers of aging [86]. In addition, gene ontology enrichment analysis of the core SASP proteins showed that they involve networks and pathways supporting age-related diseases [86]. These data suggest that the secretome of senescent cells contain aging and disease biomarkers, which travel through the circulation and systematically drive age-related dysfunctions. If this is validated, such biomarkers could be of great clinical value. 

Angptl2 was not considered in the proteomic signature of plasma aging markers [196] or in the SASP atlas [86]. However, it is important to reiterate that SASP signatures vary according to the cell type and the senescence inducer, and typical senescence markers such as p16, p21, and others considered “classical” members of the SASP are not necessarily included in the senescent signature [86,197]. Thus, the search for unknown, and the investigation of understudied senescence markers should remain a priority.

Angptl2 could display all the required characteristics to be considered as a senescent clinical biomarker of age-related diseases: (1) angptl2 is secreted by senescent cells; (2) angptl2 is a SASP protein that can be detected in human plasma; (3) plasma angptl2 levels increase with both age and age-related diseases, and angptl2 levels can be reduced with treatments. These three steps have been proposed to permit the screening of potential senescent biomarker of age-related diseases [198]. However, the potential role of angptl2 as a senescent clinical biomarker of age-related diseases is still speculative and remains to be demonstrated. Indeed, whether the systemic effects of angptl2 on distant organs play a direct role in age-related diseases is not so clear: the humoral functions and/or the local autocrine/paracrine functions of angptl2 could contribute to diseases [29,54,188] (for review, see [36]). Therefore, whether or not circulating levels of angptl2 can be used as a senescent clinical biomarker deserves further investigation. Interestingly, a relationship has been reported between circulating angptl2 levels and mortality risk in patients with age-related diseases such as diabetes [178], chronic kidney diseases [199] and renal failure [200], and various types of cancer [138,201,202,203,204]. These age-related diseases are at least partly related to cellular senescence. To classify angptl2 as a senescent clinical biomarker, it still remains to be clarified whether circulating angptl2 levels are also associated with mortality risk in the general population.

## 6. Limitations

Connecting the dots between what is known about angptl2, senescence, and age-related diseases can be challenging for multiple reasons. First, as illustrated in Table 1, although angptl2 is present in the SASP of various senescent cell types, angptl2 gene/protein expression either increases or decreases in the senescent state. With rare exceptions, these changes were never discussed in the literature, and angptl2 was simply listed along with hundreds of other factors in the SASP signature. Second, transcriptomic and proteomic analyses of the SASP do not provide angptl2 concentrations, but rather fold changes; this makes comparisons between studies difficult. Thus, these complex analyses do not permit us to estimate the homeostatic range of limits of angptl2, and in turn, do not give a range for therapeutic interventions. Finally, when considering data from circulating levels of angptl2, reported to rise in multiple age-related diseases, the fact that it is unclear whether the systemic effects of angptl2 on distant organs play a direct role in age-related diseases complicates the potential classification angptl2 as a senescent clinical biomarker. An increase in systemic angptl2 with aging and in age-related diseases could rather reflect adaptive stress response to cope with the progression of the diseases.

## 7. Conclusions

Angptl2 is an exciting, understudied SASP factor. It has been identified in a multitude of SASP signatures, in various cell types exposed to different stress stimuli, and it shares striking functional similarities with typical SASP proteins. Angptl2 displays both physiological and pathological functions involved in both the beneficial and deleterious effects of cellular senescence. Angptl2 is a circulating protein and the plasma levels of angptl2 are useful to predict and to identify multiple age-related diseases in which cellular senescence and SASP are key players. Future work is needed to firmly demonstrate whether angptl2 could be a potential clinical biomarker of senescence and age-related diseases. Angptl2 likely does not act alone, but rather in synergism with other SASP factors. Through a cross-talk in the regulation of different SASP components [205], the SASP factors may influence the action of angptl2 and vice versa. It is the timely and well-orchestrated activity of the SASP as a whole, and not the activity of a single SASP factor, that will define the impact of senescence on tissue environment, spreading to neighbouring cells and thus contribute to age-related diseases [206]. Network and enrichment analysis are required to identify the SASP members of this cross-talk and the biological processes implicated. Nonetheless, angptl2 is an interesting SASP factor with the potential to become a blood biomarker of senescence, aging, and age-related diseases.

## Figures and Tables

**Table 1:** Studies that identified angptl2 as a member of the SASP.

	Cell/Tissue	Species (Model Used)	Treatment/Senescence Inducer	Changes in Angptl2 Gene Expression	Fold Change	Methods	GEO Number	Reference
**Fibroblasts**	Primary fibroblast cell lines (WS1, WI-38 & BJ)	Homo Sapiens (in vitro)	Replicative senescence (serial passages)	↓ in senescent and quiescent fibroblasts	−0.86 range (−2.84; 1.13)	cDNA microarray	NA	[58]
	Primary fibroblast cell line (IMR90)	Homo Sapiens (in vitro)	Oncogene-induced senescence (100 nM 4-hydroxytamoxifen treatment for 0–7 days)	↓ in senescent fibroblasts	−1.63	cDNA microarray	GSE41318	[63]
	Embryonic fibroblast cell line (MRC-5)	Homo Sapiens (in vitro)	SCM-induced senescence (time course over 5 days)	↓ at three time points: day 1, day 3 & day 5 vs. day 0	D1 vs. D0 = −2.39 D3 vs. D0 = −1.93 D5 vs. D0 = −2.49	RNA-seq	NA	[64]
	Primary fibroblast cell line (IMR-90)	Homo Sapiens (in vitro)	Replicative senescence (exhaustion PDL 50–59) IR-induced senescence (PDL 25 exposed to 10 Gy)	↓ in senescent fibroblasts	RS = −1.59 IR = −1.28	RNA-seq	GSE130727	[65]
	Fibroblasts from healthy skin samples	Homo Sapiens (in vivo)	Aging (young (25–27 years) and old subjects (53–70 years)	↓ in mesenchymal fibroblasts	−1.31	single-cell RNA-seq	GSE130973	[66]
	Fibroblasts from Werner Syndrome patients Premature aging disorder	Homo Sapiens (in vivo)	Patients# A0031: 37 years old TIG-114: 36 years old WSCU01: 63 years old	↑ in WS fibroblasts (vs. healthy fibroblasts)	A0031 = +10.4 TIG-114 = +5.9 WSCU01 = +7.3	cDNA microarray	NA	[67]
	Primary fibroblast cell lines (HFF)	Homo Sapiens (in vitro)	Replicative senescence (serial passages up to PDL 74)	↑ in HFF senescent fibroblasts	PDL 16–26 = +1.38 PDL 16–74 = +2.68	RNA-seq	GSE63577	[68]
	Primary fibroblast cell lines (MRC-5, WI-38, BJ, IMR-90 & HFF)	Homo Sapiens (in vitro)	Replicative senescence (serial passages up to PDL 74)	↑ in WI-38 senescent fibroblasts (vs. early PDL)	ND angptl2 is the 63rd most up-regulated gene	RNA-seq	GSE63577	[69]
	Primary fibroblast cell line (WI-38)	Homo Sapiens (in vitro)	IR-induced senescence (PDL 25 exposed to 10 Gy)	↑ in WI-38 senescent fibroblasts	+1.15	RNA-seq	GSE130727	[65]
	Primary fibroblast cell line (IMR-90)	Homo Sapiens (in vitro)	Paracrine- and oncogene-induced senescence (4-hydroxytamoxifen treatment)	↑ in senescent fibroblasts	ND	single-cell RNA-seq	GSE115301	[70]
**Hepatocytes**	Hepatocellular carcinoma cell line (Huh7 clones)	Homo Sapiens (in vitro)	Reprogramming replicative senescence (clone C3: PDL80; clone G12: PDL 90)	↓ in senescent Huh7 clones C3 and G12	ND	cDNA microarray	GSE17546	[71]
	Hepatocellular carcinoma cell line (HepG2)	Homo Sapiens (in vitro)	Oncogene-induced senescence (10 µM and 100 µM etoposide treatment for 30 h)	↑ in hepatocytes exposed to etoposide	10 µM vs. ctrl = +3.63 100 µM vs. ctrl = +1.76 10 µM vs. 100 µM = +2.06	cDNA microarray	GSE61110	[72]
	Hepatocellular carcinoma cells from patients	Homo Sapiens (in vivo)	Oncogene-induced senescence (liver cancer)	↑ between predicted high and low risk HCC cases	high vs. low risk = +1.16	cDNA microarray	GSE14520	[73]
**Astrocytes**	Fetal astrocytes	Homo Sapiens (in vitro)	Oxidative stress-induced senescence (200 µM H_2_O_2_ treatment for 2 h)	↑ between pre-senescent and senescent astrocytes	pre vs. senescent = +2.05	RNA-seq	GSE58910	[74]
	Primary astrocyte cell line	Homo Sapiens (in vitro)	IR-induced senescence (exposed to 10 Gy)	↑ between non-senescent and senescent astrocytes	+1.21	RNA-seq	NA	[75]
**Vasculature**	Aortic smooth muscle cells	Homo Sapiens (in vitro)	Replicative senescence (serial passages growing population = PDL 14 senescent population = PDL 39–42)	↓ in VSMC from late PDL vs. early PDL	−2.8	cDNA microarray	NA	[76]
	Endothelial cell lines (HUVEC & HAEC)	Homo Sapiens (in vitro)	IR-induced senescence (exposed to 4 Gy)	↓ in senescent ECs	HAECs = −0.21	RNA-seq	GSE130727	[65]
	Pericytes from brain of young and old mice (C57Bl/6)	Mus Musculus (in vivo)	Aging (young mice: 3 months old aged mice: 28 months old)	↓ in pericytes from young to old mice brain	ND % expression: 20% (young) vs. 10% (aged)	single-cell RNA-seq	NA	[77]
	Arterial endothelial cells from aortic artery	Macaca Fascicularis (in vivo)	Replicative senescence (young monkey: 4–6 years old old monkey: 18–21 years old)	↓ in senescent aortic ECs	−0.38	single-cell RNA-seq	GSE117715	[78]
	Endothelial cells derived from embryonic stem cells	Homo Sapiens (in vitro)	Replicative senescence (serial passages in FOXO3A^−/−^ ECs)	↓ in senescent FOXO3A^−/−^ ECs	−0.87	single-cell RNA-seq	GSE117715	[78]
**Others**	Prostate and uterus (healthy samples)	Homo Sapiens (in vivo)	Aging (1-year follow-up in healthy individuals 20–79 years old)	↓ in healthy tissues upon aging (1 year)	Prostate = −0.01 Uterus = −0.02	RNA-seq	NA	[79]
	Breast and uterus tumor samples	Homo Sapiens (in vivo)	Aging (1-year follow-up in breast and uterus cancer patients)	↓ in cancerous tissues upon aging (1 year)	Breast = −1.94 Uterus = −1.77	RNA-seq	NA	[79]
	Platelets from healthy donors	Homo Sapiens (in vitro)	Replicative senescence (healthy platelets stored under standard blood banking conditions for 5 days)	↑ between day 0 and day 5	+3.00	microarray	NA	[80]
	Brain tumor samples	Homo Sapiens (in vivo)	Aging (1-year follow-up in brain cancer patients)	↑ in cancerous tissue upon aging (1 year)	+1.75	RNA-seq	NA	[79]

GEO: gene expression omnibus accession number; IR: ionizing radiation; NA: non applicable; ND: not determined; PDL: population doubling length; SCM: stem cell medium (ROS-induced oxidation). ↑: up-regulation; ↓: down-regulation.
